# What's wrong with drug development for sickle cell disease?

**DOI:** 10.1002/hem3.70082

**Published:** 2025-02-06

**Authors:** Valentine Brousse, David Rees, Raffaella Colombatti

**Affiliations:** ^1^ Centre de Référence MCGRE, Service d'Hématologie‐Immunologie, AP‐HP Hôpital Robert Debré Paris France; ^2^ Université Paris Cité and Université des Antilles, Inserm, BIGR Paris France; ^3^ Department of Paediatric Haematology King's College Hospital London UK; ^4^ Red Cell Haematology Lab, Comprehensive Cancer Centre, School of Cancer and Pharmaceutical Science King's College London UK; ^5^ Pediatric Oncology Hematology Unit, Department of Women's and Children's Health Azienda Ospedale‐Università di Padova Padua Italy; ^6^ Department of Women's and Children's Health University of Padova Padua Italy

The repeated failure to bring new disease‐modifying or curative therapies to individuals with sickle cell disease (SCD) has sadly been demonstrated yet again.

Newly marketed drugs, which were much awaited by patients with SCD and provisionally approved for use in many countries, were withdrawn because of clinical trials showing a lack of benefit and/or possible harm. More specifically, crizanlizumab, an antiadhesive monoclonal antibody failed to demonstrate meaningful clinical benefit in a confirmatory phase 3 trial and had its approval withdrawn across Europe by the EMA and equivalent authorities.^1^ More recently, in September 2024, voxelotor, an anti‐sickling agent, approved by the FDA and EMA, was abruptly withdrawn from the market and ongoing clinical trials by the manufacturer, following trial and registry data showing an unexpected excess of deaths and clinical complications in patients taking the drug.^2^ In the last 5 years, several other drugs have also been dropped after failing to show benefit in late‐stage clinical trials in SCD, including rivipansel, sevuparin, ticagrelor, prasugrel, and poloxamer 188.^3^, ^4^, ^5^, ^6^, ^7^ To date, no drug targeting acute vaso‐occlusive pain, the major debilitating and recurrent symptom of the disease, has been successfully developed. At the end of 2024, patients are left with hydroxyurea, a 150‐year‐old drug, which is effective but definitely not curative. Last but not least, effective curative treatments involving gene therapies are currently not available in Europe, despite highly promising results. So, what is wrong with drug development for SCD?

At this stage, it is not entirely clear where the blame lies. It seems possible that useful drugs have been lost to individuals with SCD because of badly designed or executed clinical trials with inappropriate endpoints, complex socioeconomic issues, or other selection biases, possibly driven by the desire of pharmaceutical companies to complete trials and access the market rapidly for economic reasons. Equally, it is possible that ineffective and potentially harmful drugs were prematurely approved for use by many authorities across the world and promoted by healthcare providers and patient associations, eager to have an additional treatment. Clearly, it is important to learn from these experiences.

First, it is vital to try and design more appropriate clinical trials. Important factors to consider include how outcomes in SCD vary very widely across different countries and continents, and that the effects of a drug in European countries, where more than 95% of children with sickle cell disease survive to adulthood, may be very different to the effects in some African countries, where fewer than 20%–50% affected children survive.^8^ Similarly, it is important to focus on appropriate endpoints, with the recent voxelotor experience perhaps illustrating that although laboratory endpoints, such as increased hemoglobin levels, are easier to achieve, it is still important to demonstrate some meaningful clinical benefit, such as reduced episodes of pain or improved quality of life.^9^ Patient‐reported outcomes in particular need to be included as ultimately they are the outcomes that matter.^10^

Importantly, drug trials are extremely costly, and academic trials for drug development are scarce, increasing reliance on pharmaceutical companies. These are wealthy enough to finance research throughout the journey from drug screening to preclinical and clinical studies, regulatory filing, and marketing. While these companies have obviously largely promoted their own interests, they have also benefited patients with SCD, by raising awareness of the condition. Importantly, pharmaceutical companies have conducted international trials in low‐income countries where the majority of patients live, potentially giving them access to otherwise unaffordable drugs, although it is unclear whether these drugs would ever be affordable in these countries if they came to market. But pharmaceutical companies are not philanthropic, and it seems possible that their interactions with experts in SCD (including ourselves) may have led to inappropriate support for drugs, which actually showed little evidence of clinical benefit in trials or real life.

It is also apparent that more needs to be invested in research to identify novel pathophysiological processes in SCD and develop new therapeutic approaches. Encouragingly, some new drugs are progressing to late‐phase clinical trials, such as the pyruvate kinase activators, and there is rapid progress in the development of stem cell treatments, including gene editing and the use of alternative‐donor transplantation. Most effective treatments seem likely to involve the safe inhibition of sickle hemoglobin polymerization, and there is also encouraging progress in this area, including novel ways to promote hemoglobin F production.^11^ As precision medicine is emerging in all fields, tailored therapy based on combined treatment addressing various pathophysiological pathways in SCD will presumably also be part of the answer in the upcoming years. Combination therapies, which are currently significantly underexplored, will require adequate trial design and data collection, interdisciplinary collaboration, and scientific involvement of various stakeholders at the same time.

There are evidently structural and systemic racial issues in drug development for SCD, which cannot be ignored and which are important in themselves, but are also related to the fact that black populations are often those with less influence, less political representation, less patient advocacy, and fewer opportunities to demand access to cure.^12^ Consequently, research in SCD has been underfunded, despite long‐established evidence that health outcomes are correlated to funding and that it is economically wise to alleviate the cost of the condition with disease‐modifying or curative therapies. Arguably, the pathophysiology of SCD is complex but cystic fibrosis is probably even more complex, and the difference in research funding, drug development, and available treatment possibilities is shockingly greater.^13^

Discussions between experts, patients, funding agencies, universities, pharmaceutical companies, regulatory agencies, and other stakeholders are crucial in rare diseases like SCD. There needs to be increased funding, a better combination of academic and industry‐based studies, early involvement of patients, better clinical trials with appropriate patient populations and end‐points, and perseverance to develop effective new drugs for SCD, which is undoubtedly possible. International clinical trials that enroll patients in very different settings need to specifically address differences in socioeconomic status, healthcare access, analgesic drug availability, cultural differences, and climatic effects, to avoid the failures which affect the entire SCD community across the world. Ultimately, we also need curative treatments that are available to all patients. Experts and patients need to advocate for the life‐transforming dimension of these treatments, leaving the discussion of cost to policymakers, who must better justify the denial of innovative treatment to people with SCD while allowing costly innovative therapies for patients with malignant hematological diseases.^14^ If nothing else, we need to try harder and fail better.

## CONFLICT OF INTEREST STATEMENT

The authors declare no conflicts of interest.

## AUTHOR CONTRIBUTIONS

All authors were involved in devising, planning, writing, and revising the manuscript, and all approved the final version.

## FUNDING

This research received no funding.

## References

[1] ClinicalTrials.Gov. Study of two doses of crizanlizumab versus placebo in adolescent and adult sickle cell disease patients. Accessed January 1, 2025. https://www.clinicaltrials.gov/study/NCT03814746

[2] Pfizer Voluntarily Withdraws All Lots of Sickle Cell Disease Treatment OXBRYTA® (voxelotor) From Worldwide markets [press release]. Pfizer; 25 September 2024. https://www.pfizer.com/news/press-release/press-release-detail/pfizer-voluntarily-withdraws-all-lots-sickle-cell-disease

[3] Dampier C , Telen M , Wun T , et al. Randomized clinical trial of the efficacy and safety of rivipansel for sickle cell vaso‐occlusive crisis (VOC). Dampier Blood. 2023;141(2):168‐179. 10.1182/blood.2022015797 35981565

[4] Biemond BJ , Tombak A , Kilinc Y , et al. Sevuparin for the treatment of acute pain crisis in patients with sickle cell disease: a multicentre, randomised, double‐blind, placebo‐controlled, phase 2 trial. Lancet Haematol. 2021;8(5):e334‐e343. 33894169 10.1016/S2352-3026(21)00053-3

[5] Heeney MM , Abboud MR , Githanga J , et al. Ticagrelor vs placebo for the reduction of vaso‐occlusive crises in pediatric sickle cell disease: the HESTIA3 study. Blood. 2022;140(13):1470‐1481. 35849650 10.1182/blood.2021014095 PMC9523370

[6] Heeney MM , Hoppe CC , Abboud MR , et al. A multinational trial of prasugrel for sickle cell vaso‐occlusive events. N Engl J Med. 2016;374(7):625‐635. 26644172 10.1056/NEJMoa1512021

[7] Casella JF , Barton BA , Kanter J , et al. Effect of poloxamer 188 vs placebo on painful vaso‐occlusive episodes in children and adults with sickle cell disease: a randomized clinical trial. JAMA. 2021;325(15):1513. 33877274 10.1001/jama.2021.3414 PMC8058640

[8] Ranque B , Kitenge R , Ndiaye DD , et al. Estimating the risk of child mortality attributable to sickle cell anaemia in sub‐Saharan Africa: a retrospective, multicentre, case‐control study. Lancet Haematol. 2022;9(3):e208‐e216. 35240076 10.1016/S2352-3026(22)00004-7

[9] De Luna G , Habibi A , Moutereau S , et al. Fetal hemoglobin decrease during voxelotor treatment. De Luna Eur J Haematol . Published online October 21, 2024. 10.1111/ejh.14332 PMC11707809 39428915

[10] De Wit M , Cooper C , Reginster J‐Y . Practical guidance for patient‐centred health research. Lancet. 2019;393(10176):1095‐1096. 30894262 10.1016/S0140-6736(19)30034-0

[11] Gibson JS , Rees DC . Emerging drug targets for sickle cell disease: shedding light on new knowledge and advances at the molecular level. Expert Opin Ther Targets. 2023;27(2):133‐149. 36803179 10.1080/14728222.2023.2179484

[12] Lee L , Smith‐Whitley K , Banks S , Puckrein G . Reducing health care disparities in sickle cell disease: a review. Public Health Rep. 2019;134(6):599‐607. 31600481 10.1177/0033354919881438 PMC6832089

[13] Farooq F , Mogayzel PJ , Lanzkron S , Haywood C , Strouse JJ . Comparison of US federal and foundation funding of research for sickle cell disease and cystic fibrosis and factors associated with research productivity. JAMA Network Open. 2020;3(3):e201737. 32219405 10.1001/jamanetworkopen.2020.1737 PMC12549141

[14] Rouce RH , Porteus MH . Cell and gene therapy accessibility. Science. 2024;385(6708):475. 39088615 10.1126/science.ads0252

